# Expression and prognosis analysis of *DNMT* family in acute myeloid leukemia

**DOI:** 10.18632/aging.103520

**Published:** 2020-06-26

**Authors:** Ting-Juan Zhang, Liu-Chao Zhang, Zi-Jun Xu, Jing-Dong Zhou

**Affiliations:** 1 Department of Hematology, Affiliated People’s Hospital of Jiangsu University, Zhenjiang, Jiangsu, People’s Republic of China; 2 Zhenjiang Clinical Research Center of Hematology, Zhenjiang, Jiangsu, People’s Republic of China; 3 The Key Lab of Precision Diagnosis and Treatment in Hematologic Malignancies of Zhenjiang, Zhenjiang, Jiangsu, People’s Republic of China; 4 Department of Medical Laboratory, Shanghai Deji Hospital, Qingdao University, Shanghai, People’s Republic of China; 5 Laboratory Center, Affiliated People’s Hospital of Jiangsu University, Zhenjiang, Jiangsu, People’s Republic of China

**Keywords:** DNMTs, expression, prognosis, HSCT, AML

## Abstract

DNA methyltransferases (*DNMTs*) by regulating DNA methylation play crucial roles in the progression of hematologic malignancies, especially for acute myeloid leukemia (AML). Accumulating investigations have identified the high incidence of *DNMT3A* mutation in AML, and it is correlated with poor prognosis. Although a few studies have shown the expression of *DNMTs* and their clinical significance in AML, the results remain to be discussed. Herein, we systemically analyzed the *DNMTs* expression and their relationship with clinic-pathological features and prognosis in AML patients. *DNMTs* expression especially for *DNMT3A*/*3B* was closely associated with AML among various human cancers. *DNMT3A* expression was increased in AML patients, whereas *DNMT3B* expression was decreased. Significant associations between *DNMT3A*/*B* expression and clinic-pathological features/gene mutations were observed. Kaplan-Meier analysis showed that *DNMT3A* expression was associated with better overall survival (OS) and leukemia-free survival (LFS) among whole-cohort AML, and independently affected OS determined by Cox repression multivariate analysis. Notably, patients that received hematopoietic stem cell transplantation (HSCT) showed significantly better OS and LFS in *DNMT3A* lower-expressed groups, whereas patients in *DNMT3A* higher-expressed groups did not. By bioinformatics analysis, *DNMT3A* expression was found to be positively correlated with several leukemia-associated genes/microRNAs, and *DNMT3A* was identified as direct targets of *miR-429* and *miR-29b* in AML. Collectively, our study demonstrated that *DNMT3A*/*3B* showed significant expression differences in AML. *DNMT3A* expression acted as a potential prognostic biomarker and may guide treatment choice between chemotherapy and HSCT in AML.

## INTRODUCTION

DNA methylation, as the most common epigenetic modification, plays a crucial role in tissue- and stage-specific gene regulation, genomic imprinting, and X-chromosome inactivation, and has shown to be essential for normal mammalian development [1]. Also, accumulating studies have proved that both global DNA hypomethylation and hypermethylation occur frequently in tumorigenesis [2]. The hypermethylation of CpG islands at the promoter regions is often associated with the inactivation of tumor suppressor genes (TSGs) [3]. In hematopoietic disorders, aberrant DNA hypermethylation is proved to be involved in leukemogenesis [4]. For example, Spencer et al demonstrated that CpG island hypermethylation mediated by *DNMT3A* was a consequence of acute myeloid leukemia (AML) progression [5]. In addition, aberrant DNA methylation was also regarded as a dominant mechanism in progression from myelodysplastic syndromes (MDS) to AML [6]. Furthermore, deregulated DNA methylation in MDS and AML has led to the approval for the clinical use of hypomethylating agents (HMAs) in both MDS and AML patients [7].

DNA methyltransferases (*DNMTs*) are the main key effectors of DNA methylation by catalyzing the transfer of methyl groups from S-adenosyl-lmethionine to the 5’-position of cytosine residing in the dinucleotide sequence cytosine-guanine [8]. The *DNMTs* include three major members (*DNMT1*, *DNMT3A* and *DNMT3B*), among which *DNMT3A* and *DNMT3B* catalyze cytosine methylation of mammalian genomic DNA to establish *de novo* DNA methylation patterns, whereas *DNMT1* maintains a methylation state through DNA replication [9]. Recent studies have demonstrated that *DNMTs* play vital roles in the progression of hematologic malignancies, especially AML [10]. Trowbridge et al showed that haploinsufficiency of *DNMT1* impaired leukemia stem cell (LSC) function through derepression of bivalent chromatin domains [11]. More importantly, high incidence of *DNMT3A* mutation was identified in AML and *DNMT3A* mutation correlated with poor prognosis in AML [12]. Functional studies showed that *DNMT3A* was essential for hematopoietic stem cell (HSC) differentiation and mutated *DNMT3A* initiated AML [13–14], suggesting *DNMT3A* acted as a tumor suppressor gene. Although *DNMT3B* was rarely mutated in AML [15], studies have proved that loss of *DNMT3B* accelerated MLL-AF9 leukemia progression and increased expression of *DNMT3B* in LSC delayed leukemogenesis [16–17]. A few studies have shown the expression of *DNMTs* and their clinical significance in AML, but the results remain to be discussed [18–21]. Herein, we systemically analyzed *DNMTs* expression and their relationship with clinic-pathological features and prognosis in patients with AML.

## RESULTS

### DNMTs expression associated with AML among human cancer cell lines

**Figure 1:**
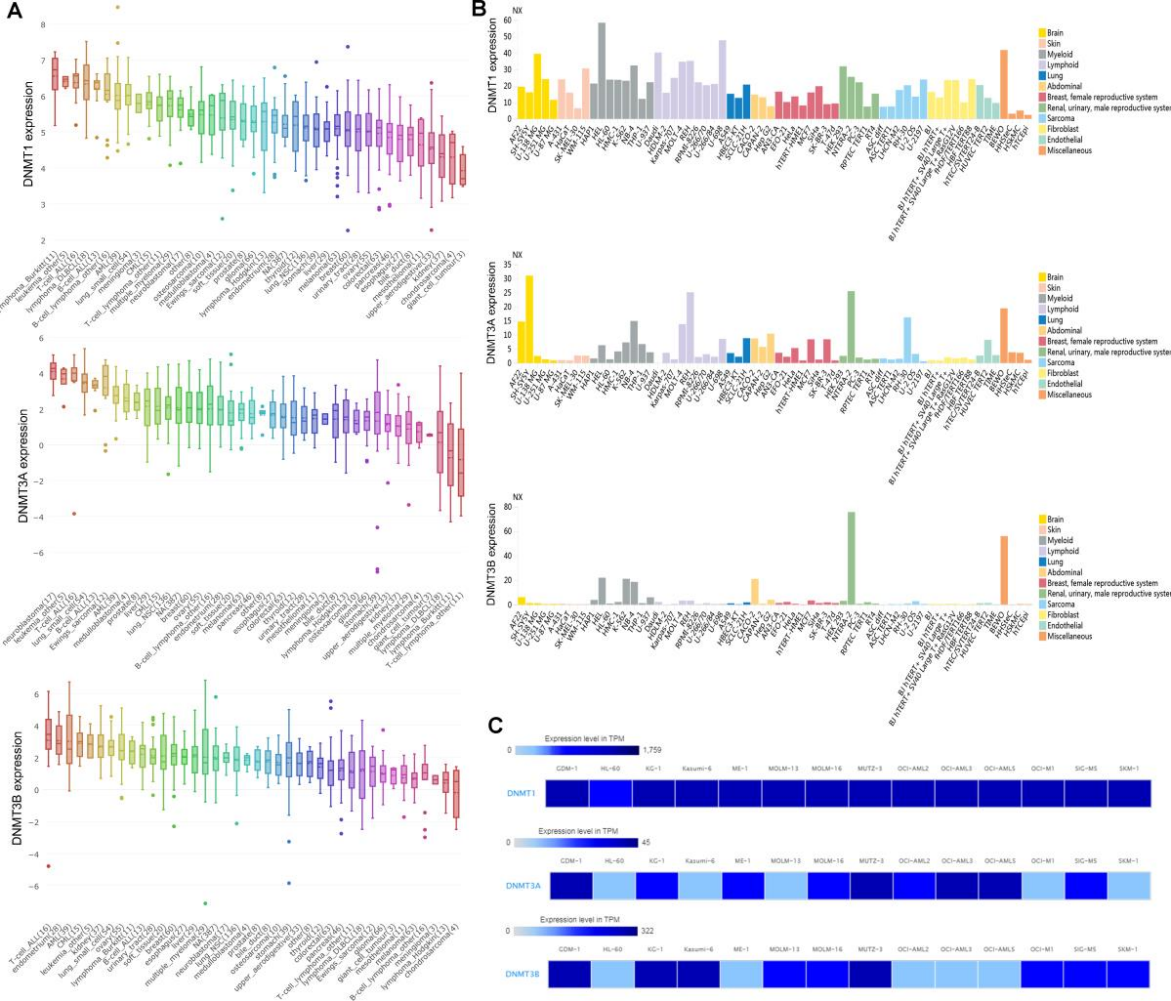
**The expression of *DNMTs* in human cancer cell lines including AML cell lines.** (**A**) The expression of *DNMTs* in leukemia cell lines, analyzing by Cancer Cell Line Encyclopedia (CCLE) dataset. (**B**) The expression of *DNMTs* in leukemia cell lines, analyzing by The Human Protein Atlas (HPA) dataset. (**C**) The expression of *DNMTs* in leukemia cell lines, analyzed by European Bioinformatics Institute (EMBL-EBI) dataset.

Using the Cancer Cell Line Encyclopedia (CCLE) databases, we found that expression of *DNMTs* was highly expressed in AML cell lines among 40 types of human cancer cell lines (Figure 1A). Moreover, *DNMTs* expression was also closely associated with myeloid cell lines revealed by The Human Protein Atlas (HPA) databases (Figure 1B). The detailed comparison of *DNMTs* expression in 14 types of AML cell lines was assessed by using the European Bioinformatics Institute (EMBL-EBI) website, which was shown in Figure 1C.

### DNMTs expression associated with AML patients among human cancers

**Figure 2:**
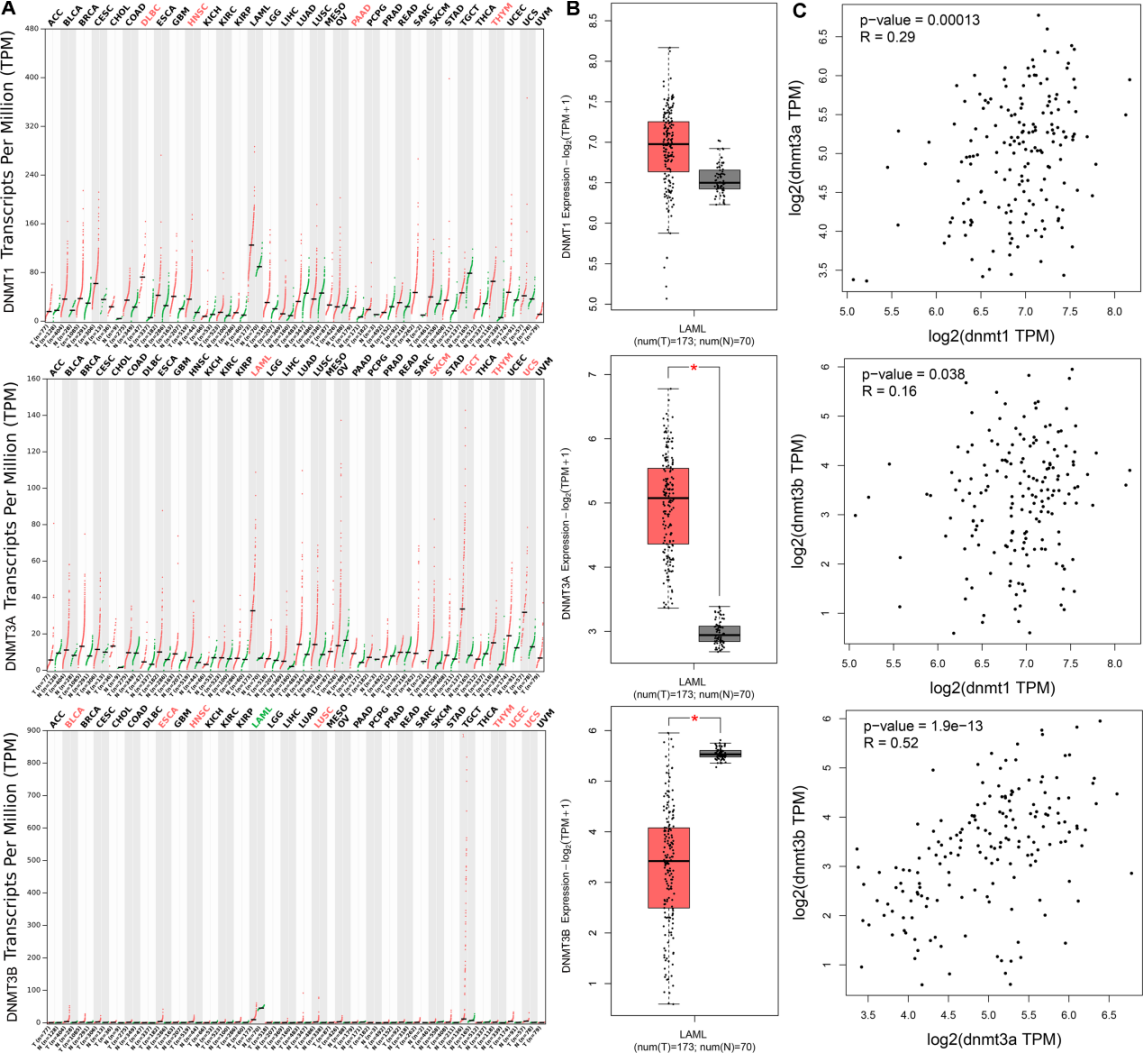
**The expression of *DNMTs* in human cancers including AML patients.** (**A**) The expression of *DNMTs* in pan-cancer analyzed by Gene Expression Profiling Interactive Analysis (GEPIA) web (http://gepia.cancer-pku.cn/). (**B**) The expression of *DNMTs* in AML analyzed by Gene Expression Profiling Interactive Analysis (GEPIA) web (http://gepia.cancer-pku.cn/). (**C**) The correction between *DNMTs* in AML analyzed by Gene Expression Profiling Interactive Analysis (GEPIA) web (http://gepia.cancer-pku.cn/).

We further determined expression of *DNMTs* in AML patients by using Gene Expression Profiling Interactive Analysis (GEPIA) dataset. Aberrant expression of *DNMT3A* and *DNMT3B* was observed in AML among 33 types of human cancers, whereas *DNMT1* did not show significant difference in AML (Figure 2A). *DNMT3A* expression was significantly increased in AML patients, whereas *DNMT3B* expression was markedly decreased in AML patients (Figure 2B). Moreover, *DNMT1* expression was slightly associated with both *DNMT3A* and *DNMT3B* expression, whereas *DNMT3A* expression was positively correlated with *DNMT3B* expression in AML patients (Figure 2C).

### Association between DNMT3A/3B expression and clinical characteristics in AML

Since aberrant expression of *DNMT3A* and *DNMT3B* was identified in AML, we further explore their clinical significance in patients with AML. Clinical implication of *DNMT3A* and *DNMT3B* was obtained by the comparison of clinical/laboratory characteristics of the AML patients between two groups divided based on median level of *DNMT3A* and *DNMT3B* transcript (Table 1). Interestingly, *DNMT3A* overexpression was associated younger age and lower white blood cells (WBCs) (*P*=0.008 and 0.063, respectively), higher peripheral blood (PB) blasts (*P*=0.006). Among the distribution of French-American-British (FAB) and cytogenetic subtypes, *DNMT3A* overexpression was associated with higher frequency of FAB-M0/M2, t(8;21), and -7/del(7) (*P=*0.009, 0.028, 0.007, and 0.007, respectively), whereas lower frequency of FAB-M4/M5 and normal karyotype (*P*=0.004, 0.001, and 0.000, respectively). In addition, *DNMT3B* underexpression was correlated with higher WBCs and lower PB blasts (*P*=0.041 and 0.006, respectively). Among the distribution of FAB and cytogenetic subtypes, *DNMT3B* underexpression was associated with higher frequency of FAB-M4/M5 and inv(16) (*P*=0.000, 0.000, and 0.001, respectively), but lower frequency of FAB-M1 and complex karyotype (*P*=0.000 and 0.000, respectively).

**Table 1:** Correlation of *DNMT3A/B* expression with clinic-pathologic characteristics in AML.

**Patient’s parameters**	***DNMT3A* expression**			***DNMT3B* expression**		
	**Low (n=87)**	**High (n=86)**	***P***	**Low (n=87)**	**High (n=86)**	***P***
Sex, male/female	47/40	45/41	0.879	47/40	45/41	0.879
Median age, years (range)	60 (18-88)	54 (21-81)	**0.008**	58 (18-88)	57 (21-81)	0.922
Median WBC, ×10^9^/L (range)	25.9 (0.8-137.2)	11.5 (0.4-297.4)	**0.063**	22.2 (1-137.2)	10.7 (0.4-297.4)	**0.041**
Median PB blasts, % (range)	17 (0-97)	48 (0-98)	**0.006**	25 (0-94)	49 (0-98)	**0.006**
Median BM blasts, % (range)	75 (30-98)	72 (32-100)	0.788	73 (30-99)	72 (30-100)	0.951
FAB classifications			**0.000**			**0.000**
M0	3	13	0.009	5	11	
M1	19	25		11	33	0.000
M2	13	25	0.028	17	21	
M3	6	10		7	9	
M4	25	9	0.004	28	6	0.000
M5	16	2	0.001	17	1	0.000
M6	1	1		0	2	
M7	3	0		1	2	
No data	1	1		1	1	
Cytogenetics			**0.000**			**0.000**
normal	55	25	0.000	42	38	
t(15;17)	6	9		7	8	
t(8;21)	0	7	0.007	6	1	
inv(16)	2	8		10	0	0.001
+8	4	4		3	5	
del(5)	1	0		0	1	
-7/del(7)	0	7	0.007	2	5	
11q23	2	1		3	0	
others	5	9		9	5	
complex	11	14		4	21	0.000
No data	1	2		1	2	
Gene mutation						
FLT3 (+/-)	28/59	21/65	0.312	22/65	27/59	0.402
NPM1 (+/-)	37/50	11/75	**0.000**	24/63	24/62	1.000
DNMT3A (+/-)	30/57	12/74	**0.002**	20/67	22/64	0.725
IDH2 (+/-)	11/76	6/80	0.307	8/79	9/77	0.804
IDH1 (+/-)	11/76	5/81	0.188	4/83	12/74	**0.038**
TET2 (+/-)	5/82	10/76	0.188	8/79	7/79	1.000
RUNX1 (+/-)	8/79	7/79	1.000	9/78	6/80	0.590
TP53 (+/-)	8/79	6/80	0.782	2/85	12/74	**0.005**
NRAS (+/-)	8/79	4/82	0.370	7/80	5/81	0.766
CEBPA (+/-)	4/83	9/77	0.162	6/81	7/79	0.782
WT1 (+/-)	4/83	6/80	0.535	4/83	6/80	0.535
PTPN11 (+/-)	5/82	3/83	0.720	4/83	4/82	1.000
KIT (+/-)	1/86	6/80	0.064	5/82	2/84	0.443
U2AF1 (+/-)	4/83	3/83	1.000	3/84	4/82	0.720
KRAS (+/-)	4/83	3/83	1.000	4/83	3/83	1.000
SMC1A (+/-)	4/83	3/83	1.000	5/82	2/84	0.443
SMC3 (+/-)	4/83	3/83	1.000	3/84	4/82	0.720
PHF6 (+/-)	3/84	2/84	1.000	3/84	2/84	1.000
STAG2 (+/-)	2/85	3/83	0.682	2/85	3/83	0.682
RAD21 (+/-)	2/85	2/84	1.000	3/84	1/85	0.621

AML: acute myeloid leukemia; WBC: white blood cells; PB: peripheral blood; BM: bone marrow; FAB: French-American-British.

### Correlation between DNMT3A/3B expression and gene mutations in AML

**Figure 3:**
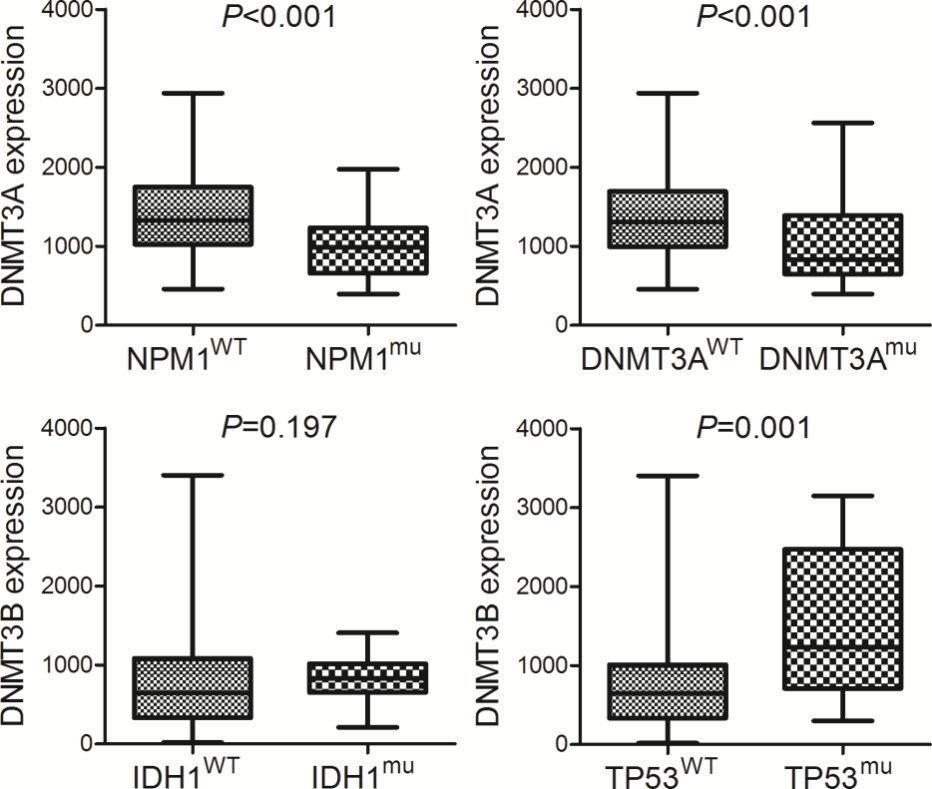
**The expression of *DNMT3A* and *DNMT3B* in AML patients with different molecular signature.** The expression of *DNMT3A* in AML patients with and without *NPM1* mutation as well as AML patients with and without *DNMT3A* mutation. The expression of *DNMT3B* in AML patients with and without *IDH1* mutation as well as AML patients with and without *TP53* mutation.

Among the common gene mutations in AML, patients with *DNMT3A* overexpression showed lower *NPM1* and *DNMT3A* mutation rates (*P*=0.000 and 0.002, respectively), whereas cases with *DNMT3B* underexpression presented lower frequency of *IDH1* and *TP53* mutation (*P*=0.038 and 0.005, respectively). In addition, we further compared the expression of *DNMT3A* and *DNMT3B* in mutation and wild-type groups of these genes (Figure 3).

### Prognostic value of DNMTs expression in AML

**Figure 4:**
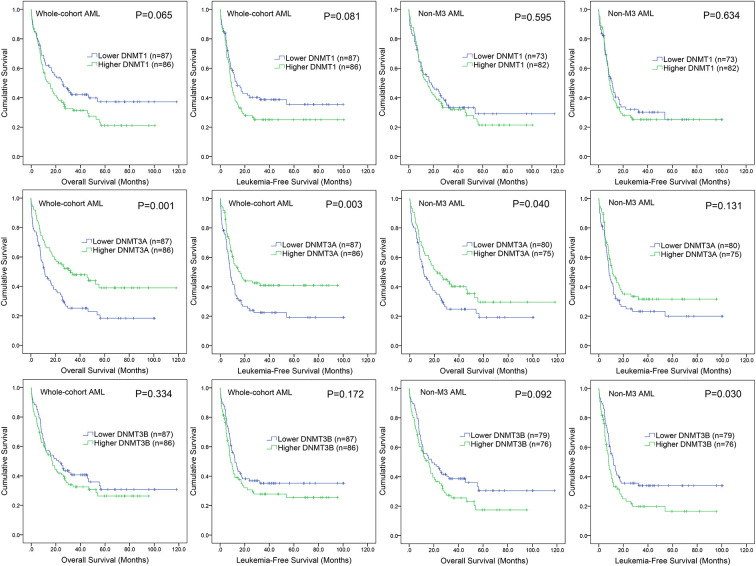
**The impact of *DNMTs* expression on survival of AML patients.** Kaplan–Meier survival curves of *DNMTs* expression on overall survival and leukemia-free survival in both chemotherapy and hematopoietic stem cell transplantation (HSCT) groups.

We next evaluate the prognostic effect of *DNMTs* expression on survival in AML. By Kaplan-Meier analysis, only *DNMT3A* overexpression was associated with longer overall survival (OS) and leukemia-free survival (LFS) in whole-cohort AML (*P*=0.001 and 0.003, respectively, Figure 4). In order to confirm the independent prognostic value of *DNMT3A* expression on both OS and LFS, we performed Cox regression analysis adjusting for prognosis-related factors. By Cox regression multivariate analysis, *DNMT3A* expression could act as an independent prognostic biomarker for OS in whole-cohort AML (Table 2). However, no significant differences were observed in either *DNMT1* or *DNMT3B* groups (Figure 4).

**Table 2:** Cox regression analyses of variables for overall survival and leukemia-free survival in AML.

**Variables**	**Overall survival**		**Leukemia-free survival**	
	**Hazard ratio (95% CI)**	***P***	**Hazard ratio (95% CI)**	***P***
*DNMT3A* expression	0.628 (0.429-0.920)	0.017	0.696 (0.476-1.020)	0.063
Age	1.038 (1.022-1.053)	0.000	1.034 (1.019-1.049)	0.000
WBC	1.008 (1.003-1.012)	0.001	1.008 (1.004-1.012)	0.000
Molecular risk	2.148 (1.537-3.000)	0.000	1.901 (1.382-2.614)	0.000
*FLT3* mutation	1.686 (1.082-2.627)	0.021	1.719 (1.100-2.687)	0.017
*CEBPA* mutation	1.685 (0.799-3.553)	0.171	1.732 (0.814-3.687)	0.154
*NPM1* mutation	0.742 (0.425-1.297)	0.295	0.810 (0.471-1.394)	0.447
*DNMT3A* mutation	1.309 (0.812-2.110)	0.269	1.134 (0.717-1.793)	0.592
*RUNX1* mutation	1.940 (1.288-2.924)	0.002	1.660 (1.104-2.498)	0.015
*TET2* mutation	0.767 (0.386-1.524)	0.448	0.824 (0.414-1.639)	0.581
*TP53* mutation	2.900 (1.483-5.669)	0.002	2.616 (1.350-5.068)	0.004
*IDH1* mutation	0.702 (0.337-1.463)	0.344	0.751 (0.344-1.639)	0.472
*IDH2* mutation	0.644 (0.338-1.226)	0.180	0.649 (0.344-1.225)	0.183
*ASXL1* mutation	1.779 (0.503-6.289)	0.372	1.813 (0.509-6.459)	0.359

AML: acute myeloid leukemia; CI: confidence interval; WBC: white blood cells. Variables in multivariate analysis including *DNMT3A* expression (low vs. high), age, WBC, karyotype (favorable vs. intermediate vs. poor), and gene mutations (mutant vs. wild-type).

### DNMT3A expression may guide treatment choice between chemotherapy and HSCT

**Figure 5:**
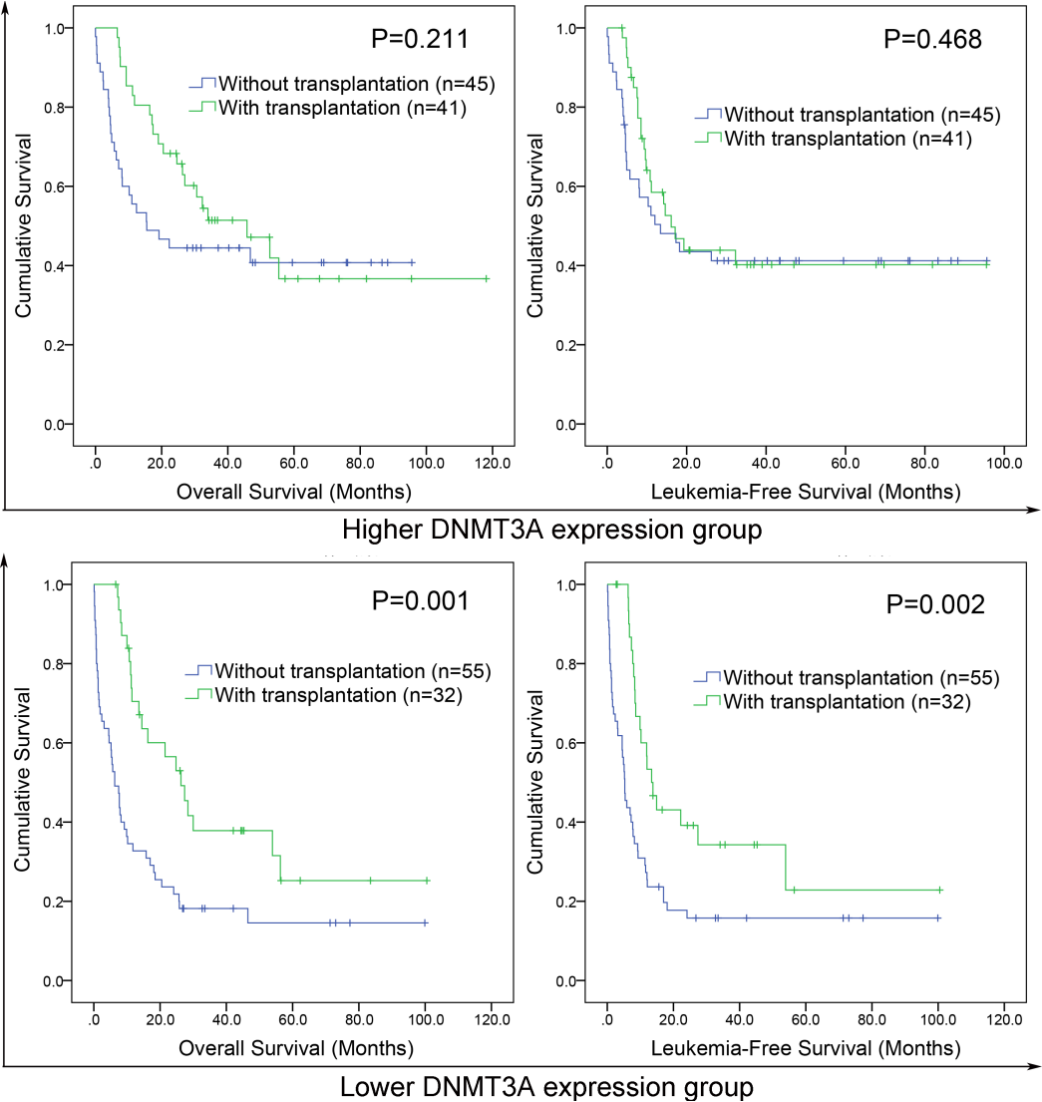
**The effect of hematopoietic stem cell transplantation (HSCT) on survival of AML patients among different *DNMT3A* expression groups.** Kaplan–Meier survival curves of overall survival and leukemia-free survival in low and high *DNMT3A* expression group.

Because lower *DNMT3A* expression predicted poor clinical outcome in AML, we intended to investigate whether patients with lower *DNMT3A* expression could benefit from HSCT. We compared OS and LFS between patients with and without HSCT in both lower and higher *DNMT3A* expression groups. In lower *DNMT3A* expression groups, patients who received HSCT showed significantly longer OS and LFS than patients who were not treated with HSCT among both total AML (Figure 5). However, in higher *DNMT3A* expression groups, there were no significant differences in OS and LFS between two groups (Figure 5). Taken together, AML patients with lower *DNMT3A* expression could benefit from HSCT, whereas those with higher *DNMT3A* expression did not. Therefore, we deduced that *DNMT3A* expression pattern may guide treatment choice between chemotherapy and HSCT.

### Molecular signature correlated with DNMT3A expression in AML

**Figure 6:**
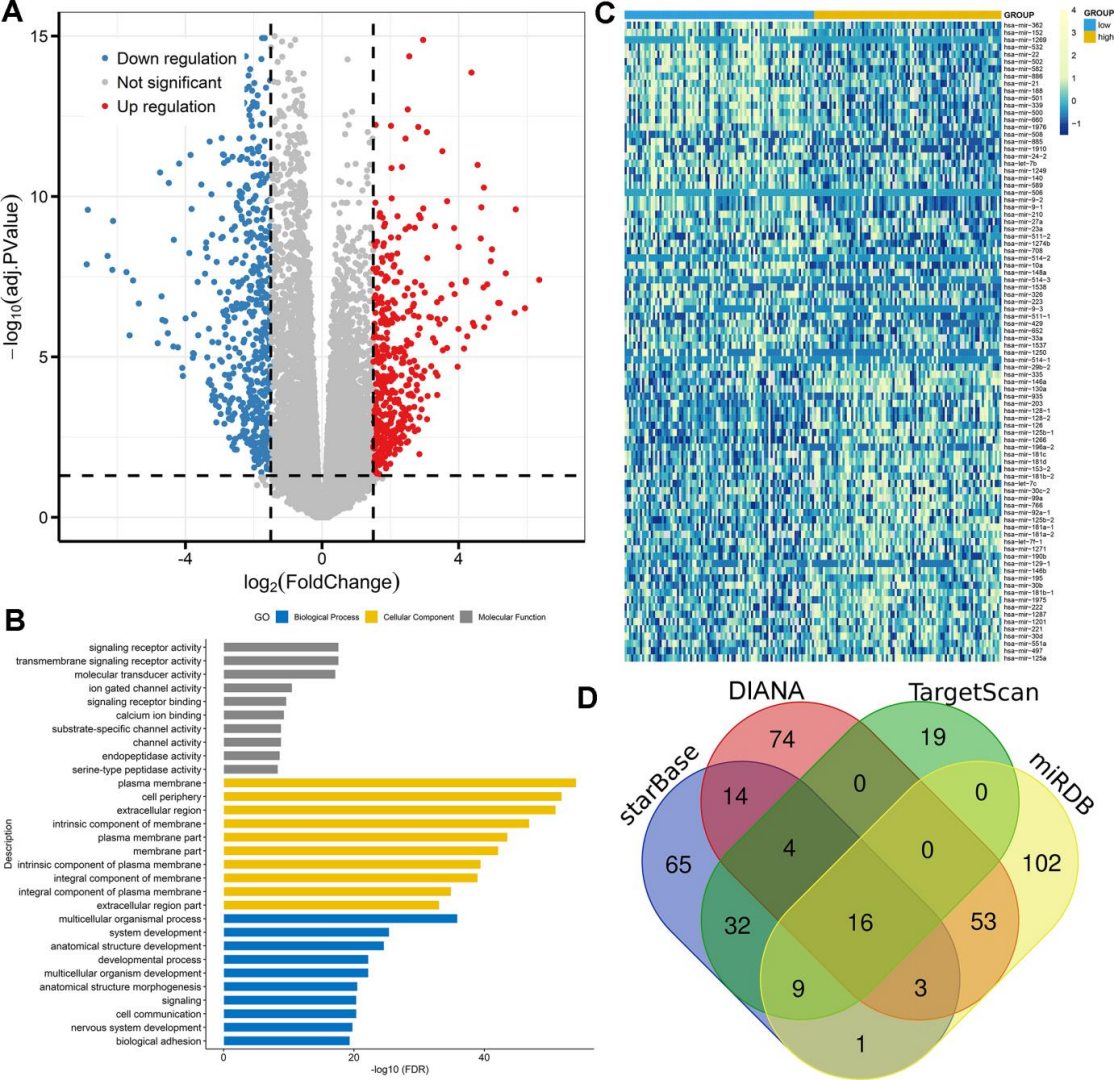
**Molecular signatures associated with *DNMT3A* in AML.** (**A**) Volcano plot of differentially expressed genes between AML patients with low and high *DNMT3A* expression (FDR<0.05, *P*<0.05, and |log2 FC|>1.5). (**B**) Gene Ontology analysis of DEGs conducted using online website of STRING (http://string-db.org). (**C**) Expression heatmap of differentially expressed microRNAs between AML patients with low and high *DNMT3A* expression (FDR<0.05 and *P*<0.05). (**D**) Venn results of microRNAs which could target *DNMT3A* predicted by DIANA (http://diana.imis.athena-innovation.gr/DianaTools/index.php?r=microT_CDS/index), miRDB (http://mirdb.org/miRDB/), TargetScan (http://www.targetscan.org/vert_72/), miRDB (http://mirdb.org/), and starBase (http://www.sysu.edu.cn/403.html).

To gain insights into the biological function of *DNMT3A* in AML, we first compared the transcriptomes of lower and higher *DNMT3A* expression groups. A total of 972 differentially expressed genes (DEGs) were identified including 428 positively correlated genes and 544 negatively correlated genes (FDR<0.05, *P*<0.05, |log2 FC|>1.5; Figure 6A; Supplementary Table 1). Positively correlated genes such as *MN1*, *BAALC*, *CD34*, and *H19* have been reported as proto-oncogenes in AML [22–24]. Furthermore, the Gene Ontology (GO) analysis was also showed in Figure 6B.

We next compared microRNA expression signature between high and low *DNMT3A* expression groups. A total of 88 differentially expressed microRNAs were identified consisting of 40 positively correlated microRNAs and 48 negatively correlated microRNAs (FDR<0.05, *P*<0.05; Figure 6C; Supplementary Table 1). The most positively correlated microRNAs such as *miR-335*, *miR-146a*, *miR-130a*, and *miR-126* were seen as oncogenic microRNAs in AML [25–28]. Moreover, negatively correlated microRNAs including *miR-22*, *miR-29b*, *miR-9*, and *miR-429* were reported as anti-leukemia roles in AML biology [29–32]. Of these negatively correlated microRNAs, *miR-29b* and *miR-429* were identified as the predicted microRNAs that could target *DNMT3A* directly (Figure 6D, Supplementary Table 2). Obviously, further studies are required to confirm the direct connections between *DNMT3A* and *miR-429* by luciferase assay.

## DISCUSSION

In this study, we systemically analyzed *DNMTs* expression and their relationship with clinic-pathological features and prognosis in patients with AML. We found that *DNMT3A* expression was increased in AML, whereas *DNMT3B* expression was decreased in AML. Although previous study showed *DNMTs* overexpression and its negative prognostic effects in AML [18–21], recent researches revealed that *DNMT3B* expression was decreased in AML blasts, whereas *DNMT1* and *DNMT3A* expression showed no significant differences [16, 33]. Additionally, the potential roles of *DNMT3A* and *DNMT3B* in AML remained poorly defined. Peters et al previously showed the tumor suppressor functions of *DNMT3A* and *DNMT3B* in the prevention of malignant mouse lymphopoiesis, but not in the development of myeloid malignancies [34]. However, ectopic *DNMT3B* expression was reported to delay leukemogenesis [17], and the loss of *DNMT3B* accelerated MLL-AF9 leukemia progression [16]. These studies demonstrated that *DNMT3B* played a crucial role in AML development, but may not act as a cancer-related driver gene during leukemogenesis. As for *DNMT3A*, it was indicated that *DNMT3A* loss progressively impaired HSC differentiation [13]. Notably, loss of *DNMT3A* and endogenous *KRAS*^G12D/+^ cooperated to regulate hematopoietic stem and progenitor cell functions in leukemogenesis [35]. In our study, we also observed the significant associations of *DNMT3A* expression with other molecular events such as *NPM1* and *DNMT3A* mutations. These studies suggested that *DNMT3A* generally not worked independently in the development of AML, and it may cooperate with other molecular events.

The prognostic value of *DNMT3A* mutation in AML has been systemically revealed. Increasing studies showed that *DNMT3A* mutations were independently associated with poor outcome in AML patients with an intermediate-risk cytogenetic profile or CN-AML [12, 36]. Moreover, loss-of-function of *DNMT3A* caused by mutations or underexpression predicted response to the HMAs decitabine treatment in AML [37]. In this study, low *DNMT3A* expression was observed to act as an independent prognostic biomarker in AML and also helpful for the selecting treatment choice between chemotherapy and HSCT. Interestingly, although two recent reports showed that high *DNMT3B* expression was a poor prognostic biomarker in AML [19, 20], we did not observe the association of aberrant *DNMT3B* expression with AML survival. The differences may be caused by the specific cell population selection and different ethnics. Obviously, prospective studies are needed to confirm and expand our results before *DNMT3A* expression pattern can be used routinely as a potential prognostic biomarker guiding treatment choice for newly diagnosed AML.

Despite that the role of *DNMT3A* in regulation of DNA methylation is well-known, the potential mechanism regulating *DNMT3A* was poorly investigated. Jost et al reported that aberrant DNA hypermethylation within the *DNMT3A* gene was frequently observed in AML, and was associated with downregulation of *DNMT3A* mRNA transcript 2 [38]. Moreover, *DNMT3A* was also identified as a direct target of a number of microRNAs such as *miR-30a-3p*, *miR-133a-3p*, *miR-450*, *miR-29a/b/c,* and *miR-129-5p* [39–43]. From our study, we observed the direct association of two microRNAs *miR-29b* and *miR-429* with *DNMT3A* in AML. Although several studies showed the direct link between *DNMT3A* and *miR-29b* in other human cancers, little studies showed the direct correlation between *miR-429* and *DNMT3A* in any type of human cancers. Interestingly, our pervious study disclosed that *miR-429* expression was decreased in AML [32], which presented the opposite expression pattern of *DNMT3A* in AML. Accordingly, further studies are required to confirm the direct associations of *DNMT3A* with *miR-429* by luciferase assay.

In summary, although we analyzed the expression and prognosis analysis of *DNMTs* expression only by public databases, our study demonstrated that *DNMT3A* and *DNMT3B* showed significant expression differences in AML. Moreover, *DNMT3A* expression acted as a potential prognostic biomarker and may guide treatment choice between chemotherapy and HSCT in AML.

## MATERIALS AND METHODS

### CCLE, HPA, and EMBL-EBI dataset

*DNMTs* expression in human cancer cell lines were assessed by the Cancer Cell Line Encyclopedia (CCLE) dataset (https://www.broadinstitute.org/ccle) [44] and also evaluated by The Human Protein Atlas (HPA) dataset (https://www.proteinatlas.org/) [45]. Moreover, *DNMTs* expression in AML cell lines was verified by the European Bioinformatics Institute (EMBL-EBI) dataset (https://www.ebi.ac.uk) [46].

### GEPIA dataset

*DNMTs* expression in AML patients and normal controls was analyzed by the Gene Expression Profiling Interactive Analysis (GEPIA) web (http://gepia.cancer-pku.cn/), whose data obtained from The Cancer Genome Atlas (TCGA) and the Genotype-Tissue Expression (GTEx) projects [47].

### Patients from TCGA datasets

A total of 173 AML patients with available *DNMTs* expression data from TCGA were identified and included in this study [15]. Clinical and molecular characteristics were collected including age, sex, white blood cell (WBC) counts, peripheral blood (PB) blasts, bone marrow (BM) blasts, French-American-British (FAB) subtypes and the frequencies of genetic mutations. After induction chemotherapy, consolidation treatment included chemotherapy (100 patients received) and hematopoietic stem cell transplantation (HSCT) (73 patients received).

### Bioinformatics analyses

The details for the identification of microRNAs targeting *DNMT3A* were reported as our previous study [48, 49].

### Statistical analyses

SPSS 22.0 were used for statistical analyses and figures creation. Mann-Whitney’s *U* test was used for the comparison of continuous variables, whereas Pearson Chi-square analysis or Fisher exact test was applied for the comparison of categorical variables. The prognostic effect of *DNMTs* expression was evaluated using leukemia-free survival (LFS) and overall survival (OS) analyzed though Kaplan-Meier analysis and Cox regression analysis. The two-tailed *P* value < 0.05 in all statistical analyses was defined as statistically significant.

### Ethical approval and consent to participate

The present study approved by the Ethics Committee and Institutional Review Board of the Affiliated People’s Hospital of Jiangsu University. Written informed consents were obtained from all enrolled individuals prior to their participation.

## Supplementary Material

Supplementary Table 1

Supplementary Table 2
